# Diagnostic and Management Hurdles in Pelvic Osteomyelitis

**DOI:** 10.7759/cureus.92655

**Published:** 2025-09-18

**Authors:** Abena K Agyekum, Nana Osei, Godslove Bonnah, Kevin Quirk, Suzette Graham-Hill

**Affiliations:** 1 Internal Medicine, State University of New York Downstate Health Sciences University, Brooklyn, USA; 2 Cardiology, Kings County Hospital Center, Brooklyn, USA

**Keywords:** advanced imaging, multidisciplinary management, pelvic osteomyelitis, prostate cancer, pubovesical fistula, radiation therapy

## Abstract

Pelvic osteomyelitis (PO) is a rare, debilitating bone infection. It often arises insidiously, posing diagnostic and therapeutic challenges. We present a 74-year-old male patient with a history of diabetes mellitus, hypertension, and prostate cancer post-radiation therapy (RT), who presented with dysuria, groin swelling, and inner thigh pain. Imaging revealed a pubovesical fistula, small retropubic abscesses, and findings consistent with PO. Prior cultures showed extended-spectrum beta-lactamase (ESBL) *Klebsiella pneumoniae* and *Candida glabrata*. Despite the patient’s refusal of urinary diversion, a six-week course of ertapenem and caspofungin resulted in clinical improvement and negative repeat cultures.

This case highlights the importance of considering PO in patients with risk factors, the critical role of advanced imaging in diagnosis, and the multidisciplinary approach necessary for effective management. While conservative antibiotic therapy succeeded here, surgical intervention is often required for long-term resolution.

## Introduction

Osteomyelitis is a severe bone infection, primarily affecting long bones, characterized by progressive inflammation and local bone destruction [1]. While the condition is most commonly associated with hematogenous spread of microorganisms, it can also arise from contiguous spread from adjacent infectious foci or direct inoculation. Pathologically, acute osteomyelitis is marked by neutrophilic infiltrates and diminished vascular integrity, while necrosis is the hallmark of chronic cases. 

Radiation therapy (RT) is a cornerstone of prostate cancer treatment, effectively targeting malignant cells through DNA damage but also affecting surrounding healthy tissues. This collateral damage can result in inflammation, vascular injury, and fibrosis, leading to various complications such as cystitis, urethral fistulas, and erectile dysfunction [2]. However, pelvic osteomyelitis (PO) is an uncommon and underreported sequela of RT. Patients with prostate cancer undergoing RT can experience a range of complications including osteomyelitis of the pelvis, which can be chronic and debilitating [3]. 

In this case report, we present a 74-year-old male patient with a history of prostate cancer post-RT, who developed PO. This case highlights the diagnostic and therapeutic challenges of PO, an often debilitating condition that requires a high clinical index of suspicion. By emphasizing the role of advanced imaging and multidisciplinary management, this report underscores the importance of considering PO in the differential diagnosis for patients with pelvic symptoms and a history of pelvic irradiation.

This article was previously presented as a meeting abstract at the 2024 New York American College of Physicians Annual Scientific Meeting on October 26, 2024.

## Case presentation

A 74-year-old male patient with a medical history of insulin-dependent diabetes mellitus, hypertension, hyperlipidemia, and prostate cancer status post-RT presented with a three-day history of dysuria, inner thigh pain, and groin swelling. He denied associated symptoms, including fevers, chills, nausea, vomiting, diarrhea, chest pain, or shortness of breath.

**Figure 1:**
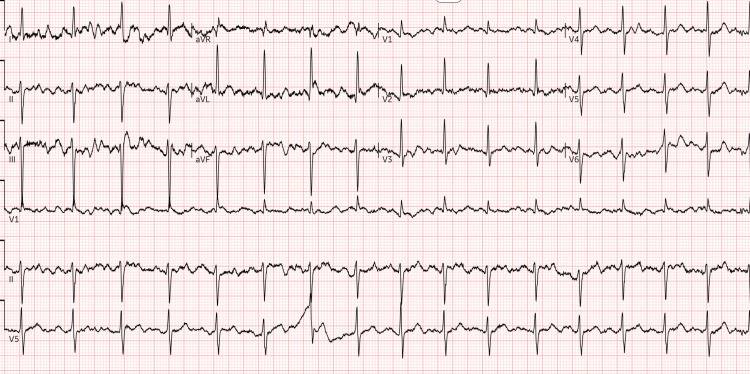
EKG strip showing left anterior fascicular block EKG: Electrocardiogram

Laboratory evaluation revealed elevated inflammatory markers, including an erythrocyte sedimentation rate (ESR) of 62 mm/hr and a C-reactive protein (CRP) level of 160 mg/L. Testing also showed poorly controlled diabetes with a hemoglobin (HB) A1c (HbA1c) of 9.5%. The white blood cell (WBC) count was 11.7 × 10⁹/L with 75% neutrophils (Table 1). Urinalysis was positive for infection. Electrocardiogram (EKG) showed left anterior fascicular block that was unchanged from prior results several months ago (Figure 1). Bilateral lower extremity Doppler ultrasonography incidentally identified a deep vein thrombosis (DVT) in the superior right superficial femoral vein.

**Table 1:** Laboratory values comparing values on Days 1, 7, and 14 of treatment WBC: White blood cells, HB: Hemoglobin, PLT: Platelets, RBG: Random blood glucose

Parameter	Day 1	Day 7	Day 14	Reference
WBC	11.73	9.66	5.56	4.50-10.90 K/uL
HB	7.6	7.8	7.8	14.0-18.0 g/dL
PLT	533	512	567	130-400 k/uL
RBG	327	136	143	70-99 mg/dL
Anionic gap	11	10	11	5-15 mEq/L
Sodium	131	134	136	136-146 mmol/L
Potassium	4.2	4.4	4.4	3.5-5.0 mmol/L
Chloride	96	102	105	98-106 mmol/L
Bicarbonate	24	22	20	24-31 mmol/L
Creatinine	1.57	1.79	14.8	0.70-1.20 mg/dL
Blood urea	34.0	26.0	29.0	8.0-23.0 mg/dL

CT of the abdomen and pelvis revealed a pubovesical fistula with a small abscess containing gas at the pubic symphysis. There was also interval progression of bladder wall thickening and the presence of a large focus of gas within the bladder. Due to the erosive changes at the pubic symphysis, differential diagnoses included osteomyelitis and septic arthritis.

**Figure 2:**
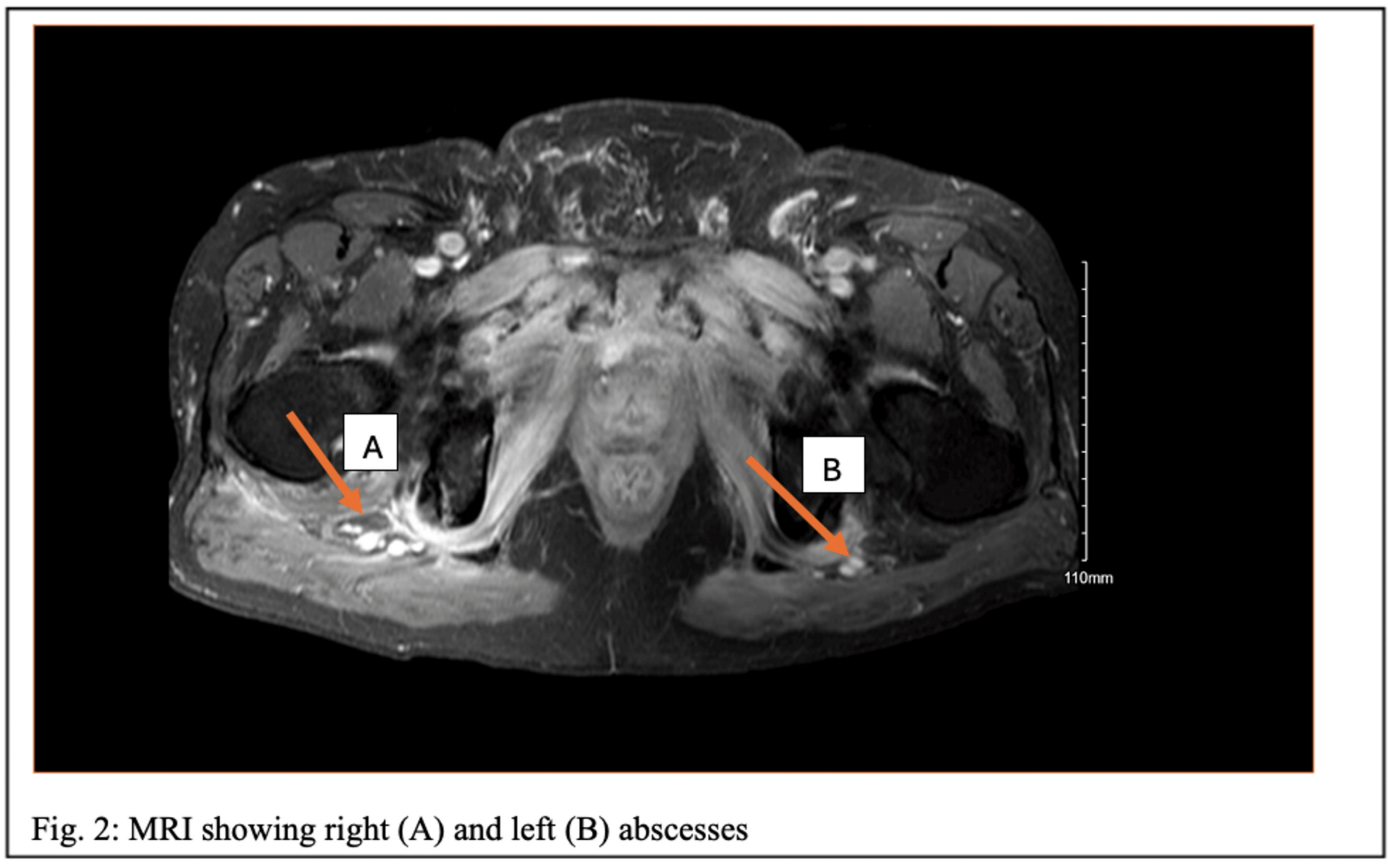
Axial T1-weighted MRI postcontrast showing right and left pelvic abscesses

MRI of the pelvis revealed findings consistent with acute osteomyelitis involving the pubic body and bilateral inferior pubic rami and ischial tuberosities. Phlegmonous changes extended from the pubic symphysis to the retropubic space of Retzius, pelvic sidewalls, and ischial tuberosities. Retropubic abscesses measured up to 4.7 cm in the right retropubic space and 1.4 cm in the left retropubic space. Additional collections surrounded the bilateral ischial tuberosities, each measuring up to 1.9 cm (Figure 2). 

A review of prior medical records revealed that the patient had similar abscesses drained at another facility several months earlier. Urine cultures from that episode grew extended-spectrum beta-lactamase (ESBL) *Klebsiella pneumoniae* and *Candida glabrata*. Imaging at that time demonstrated smaller abscesses, which have since increased. However, the patient had left the hospital against medical advice before completing treatment.

During this admission, interventional radiology was consulted but deemed the abscesses unsuitable for drainage due to their small size and challenging anatomical positioning. The urology team recommended urinary diversion with Foley catheterization, which the patient declined. The infectious disease team initiated a six-week regimen of ertapenem and caspofungin based on prior culture sensitivities, resulting in marked clinical and laboratory improvement. The incidental DVT was treated with full-dose anticoagulation. His poorly controlled diabetes was managed with insulin, achieving better glucose control during hospitalization.

The patient was discharged with a peripherally inserted central catheter (PICC) line to complete intravenous antibiotics at home. He was scheduled for outpatient follow-up with his primary-care physician, endocrinology, infectious disease, and urology. Close monitoring with serial imaging and possible surgical intervention were recommended to address the underlying pathology.

## Discussion

Osteomyelitis of the pelvis remains a diagnostic challenge due to its rarity, accounting for only 1-11% of all osteomyelitis cases [4]. Few documented cases exist, and one notable instance involves a 39-year-old woman who developed pubic bone osteomyelitis following childbirth. Her symptoms were subtle, primarily presenting as localized pelvic pain and fever [5]. PO often presents insidiously, mimicking other conditions such as septic arthritis. As a result, a high index of clinical suspicion is crucial for timely diagnosis and intervention [6]. PO is typically not included in the differential diagnosis for patients with similar presentations, as illustrated in our case, which often leads to delayed diagnoses [7].

This patient exhibited subtle symptoms not immediately suggestive of a severe infection. However, his medical history of prostate cancer and prior RT were significant risk factors. Advanced imaging played a pivotal role in diagnosis, revealing findings consistent with acute osteomyelitis involving the pubic body, pubic rami, and bilateral ischial tuberosities - an atypical location for such an infection [8].

Surgical intervention was not indicated, given the small size of the abscess. The patient was treated with a six-week course of antibiotics based on culture results, with subsequent clinical improvement. It is important to note that while conservative management with antibiotics can be effective, it may often fail, and surgical intervention, such as urinary diversion and abscess drainage, may be necessary in the long term. Cosma et al. highlighted that prompt evaluation in most cases of PO typically leads to favorable outcomes, with few cases requiring surgical intervention [5].

PO typically presents with fever, chills, and localized pain. It is almost always secondary to infection, which may result from either direct inoculation of bacteria into the bone or hematogenous spread [9]. This patient presented with dysuria and pelvic pain, which could have been mistaken for a recurrence of prostate cancer given the patient's medical history. However, imaging studies, including MRI and CT, revealed otherwise. While prostate cancer can lead to osteoblastic lesions in over 90% of cases, with a small proportion presenting mixed osteoblastic and osteoclastic lesions, such findings were not evident in this case, as confirmed by imaging [10].

A potential mechanism for the development of PO in this patient post-RT could involve seeding from a pubovesical fistula, a rare complication of RT [11]. Several cases have been reported in prostate cancer patients post-RT, where osteomyelitis developed in the presence of prostatosymphyseal and pubosymphyseal urinary fistulas [11,12]. In this case, the connection between the bladder and pubic bone may have allowed infectious agents from the patient's urine cultures to inoculate the pelvis. The patient's pelvic region would have been more susceptible to infection due to potential RT-induced adverse effects, such as vascular insufficiency, which could impair an adequate immune response. Given the complexity of presentation, diagnosis, and treatment, patients with PO benefit from a multidisciplinary treatment approach, involving infectious disease specialists, urologists, general surgeons, and interventional radiologists, as demonstrated in our case [13].

## Conclusions

PO represents a diagnostic and therapeutic challenge due to its insidious presentation, overlapping symptoms with other pelvic pathologies, and anatomical complexity. This case underscores the importance of a multidisciplinary approach for managing this condition, integrating advanced imaging modalities, infectious disease expertise, and surgical or interventional input when necessary. Uncontrolled diabetes, prior RT, and recurrent infections, as seen in this patient, are critical risk factors that can complicate the disease course and influence treatment outcomes.

Effective management relies on early recognition, targeted antimicrobial therapy guided by culture sensitivities, and addressing underlying comorbidities such as glycemic control and venous thromboembolism. This case also highlights the potential for recurrence and the need for robust follow-up, including imaging and interdisciplinary care. By leveraging comprehensive clinical strategies, clinicians can improve outcomes and mitigate the significant morbidity associated with PO.
